# Mixed germ cell tumor of the testicle with ravdomuosarcomatous component: a case report

**DOI:** 10.1186/1757-1626-2-9299

**Published:** 2009-12-10

**Authors:** Konstantinos Stamatiou, Panagiotis Papadopoulos, Georgios Perlepes, Nikolaos Galariotis, Michalis Olympitis, Hippocrates Moschouris, Theodora Vasilakaki

**Affiliations:** 1 Urology Department, General Hospital of Pireas "Tzaneio", Greece; 2 Radiology Department, General Hospital of Pireas "Tzaneio", Greece; 3 Pathology Department, General Hospital of Pireas "Tzaneio", Greece

## Abstract

**Introduction:**

Testicular tumors can be classified as seminomatous and non-seminomatous germ-cell tumor (NSGCT) types. Mixed germ cell tumors contain more than one germ cell component and are much more common than any of the pure histologic forms representing 32%-60% of all germ cell tumors. The composition of these tumors varies. Here we present a rare case of a mixed germ cell tumor composed of seminoma, Yolk sack tumor and teratoma containing a sarcoma component of somatic type malignancy.

**Case presentation:**

A 32-year-old Caucasian male presented with history of right-sided scrotal swelling since 6 months. Backache was present since 2 months and a history of right epididimitis was also present since 8 months. Alpha-Fetoprotein, beta-HCG and LDH values were found abmormal. USG of the scrotum revealed a large right testis swelling characterized by scarce cystic elements and calcifications. CT scan of the abdomen showed nodular metastasis involving the interaortocaval, precaval, and right para-aortic lymph nodes. The block of enlarged lymph nodes infiltrated the psoas muscle. The patient underwent right-sided high orchidectomy and was given chemotherapy of the BEP regimen. After the 2nd cycle the patient discontinued the chemotherapy and when he came for follow-up after a gap of 3 months, despite the normalisation in tumor markers values, the retroperitoneal mass was relapsed. CT scan of the chest showed multiple lung metastases.

**Conclusion:**

More than 50% of germ-cell tumors include more than 2 basic germ-cell tumor types, with the exception of spermatocytic seminoma. About 90% of the patients with nonseminomatous tumors can achieve complete cure with aggressive chemotherapy and most of them can be cured. Although prognosis of testicular tumors depends largely on clinical stage, histological type and adhesion to the treatment influence the prognosis as well.

## Background

Testicular cancer is a respectively rare neoplasm; It make up approximately two percent of all malignant cancers in men and account for up to ten percent of all malignant disease occurring within the male genitourinary system. Most of these tumors occur in three age groups - infancy, late adolescence and early adulthood. More importantly, testis tumors are the most common malignant disease, developing in men between 20 and 40 years of age and are the third leading cause of death among men of this age group [1].

Pathologically, testis cancers are divided into two classes; germ cell tumors which are derived from germinal epithelium and non-germinal tumors which are of gonadal stroma origin. Tumors of germ cell origin comprise about 95% of all testis cancer. Germ cell tumors are divided into two basic groups: seminomas which occur in approximately 40% of the population and non-seminomatous tumors (NSGC) which may be seen in pure or mixed form [2].

NSGCs are further divided into the following five groups:

1) embryonal carcinoma with or without seminoma, which occurs in about 25% of the group;

2) teratoma with or without seminoma, which occurs in about 7% of the group;

3) teratocarcinoma including teratoma with embryonal carcinoma, choriocarcinoma, or both with or without seminoma occurring in about 25% of the group;

4) choriocarcinoma with or without seminoma or embryonal carcinoma or both account for the remaining 1-3%.

Mixed germ cell tumors contain more than one germ cell component and are much more common than any of the pure histologic forms representing 32%-60% of all germ cell tumors. Essentially, any admixture of the germ cell tumors as seen in pure form may be seen, one of the most common admixtures being embryonal carcinoma and teratoma [3]. Minor foci of yolk sac tumor are common, although it is usually overshadowed by other components, such as embryonal carcinoma. As is typical of embryonal carcinoma when seen in pure form, epithelium is often associated with syncytiotrophoblast giant cells when seen as part of a mixed germ cell tumor. Although seminoma may be seen as part of a mixed germ cell tumor, in some cases one sees seminoma separate from a dominant mass of non-seminomatous mixed germ cell neoplasia, and in such cases it is probably truly multicentric neoplasia, although for sign-out purposes it is probably sufficient to consider the seminoma together with the other neoplastic components under the one designation of mixed germ cell tumor with the traditional rough quantitation of the various components in descending order of frequency. The average age of presentation for patients with mixed germ cell tumors is 30 years. Unfortunately, many of these patients present late, usually with some or the other complications which are difficult to treat and carry bad prognosis. Still, if they can complete the chemotherapy they have a reasonable survival period, depending on the complications they have. We report on a patient who represents this unusual mixed variety of germ cell tumor.

## Case reports

A 32-year-old Caucasian male presented with history of right-sided scrotal swelling since 6 months. According to the patient, it was a right -sided painless swelling which had progressively increased in size to its present dimensions. There was heaviness in the right side of the scrotum. There was no skin involvement. Backache was present since 2 months, more on walking and on straining. A history of right epididimitis since 8 months was also present.

The patient was averagely built and nourished. Vital parameters were stable. The abdomen was soft on palpation with no obvious organomegaly. The chest was clear on auscultation. On local examination, a large right -sided scrotal swelling was found. The scrotal skin was normal. No scar, sinuses or dilated veins were seen. On palpation, local temperature was normal and testicular sensations were absent on the affected side. It was a painless, non tender, non transilluminant swelling with variegated consistency. Per-abdominal examination did not reveal any abnormality. Virchow's nodes were negative.

Hematocrit was 42,5%, WBC:7100, PLT:213000, Glu:92, Ur:41 mg/dL, Cr:0,9 mg/dL, CPK:132.

Tumor markers: Alpha-Fetoprotein (AFP):2628 ng/ml, Beta-HCG:6,96 IU/ml, LDH:979

USG of the scrotum revealed a large right testis swelling of 45 × 37 × 53 mm. The eco architecture of the affected testis was promiscuous characterized by scarce cystic elements and calcifications. No dilated veins were seen.

USG of the abdomen showed a large right retroperitoneal mass corresponding to para-aortic lymph node block.

**Figure 1:**
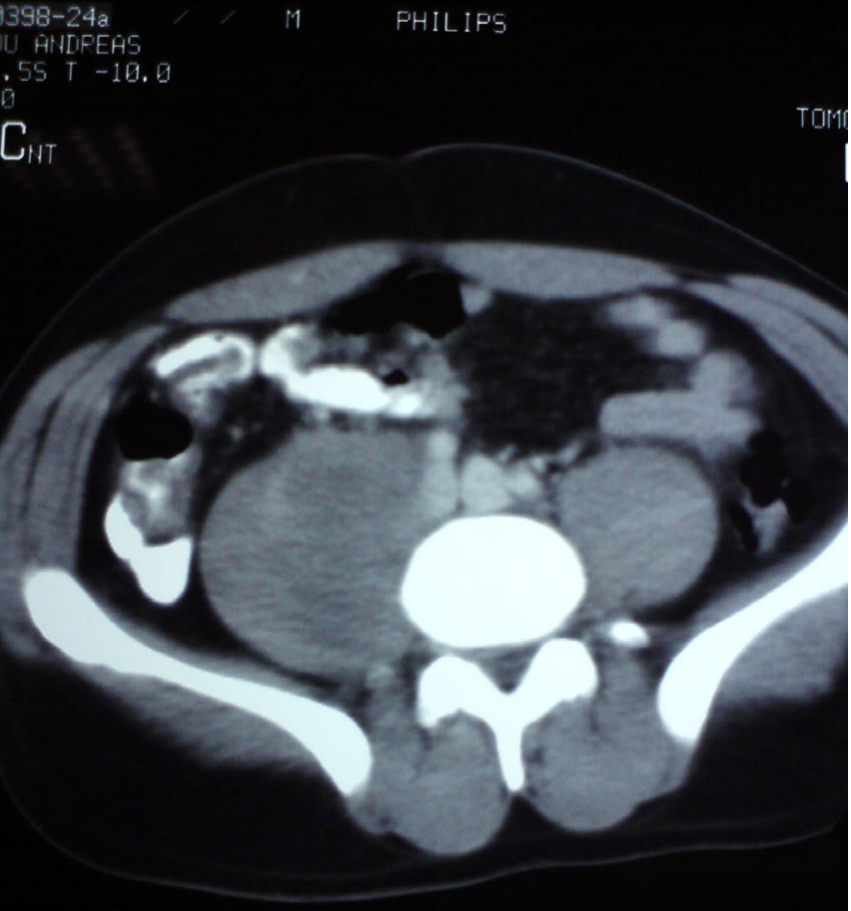
**Nodular metastasis involving the interaortocaval, precaval, and right paraaortic lymph nodes**.

**Figure 2:**
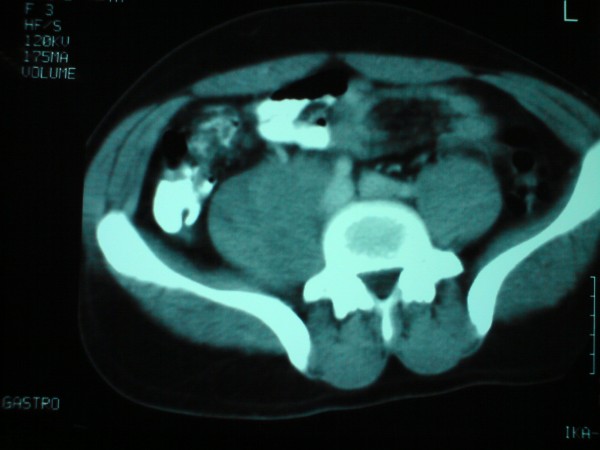
**Block of enlarged lymph node filling almost the entire right retroperitoneal space infiltrating the psoas muscle**.

CT scan of the abdomen showed nodular metastasis involving the interaortocaval, precaval, and right paraaortic lymph nodes. The block of enlarged lymph node was filling almost the entire right retroperitoneal space infiltrating the psoas muscle (figures 1 and 2). Liver, pancreas, kidneys and spleen were found normal.

Both Chest x-Ray and CT scan of the chest showed no lymphadenopathy.

The patient underwent right-sided high orchidectomy.

**Figure 3:**
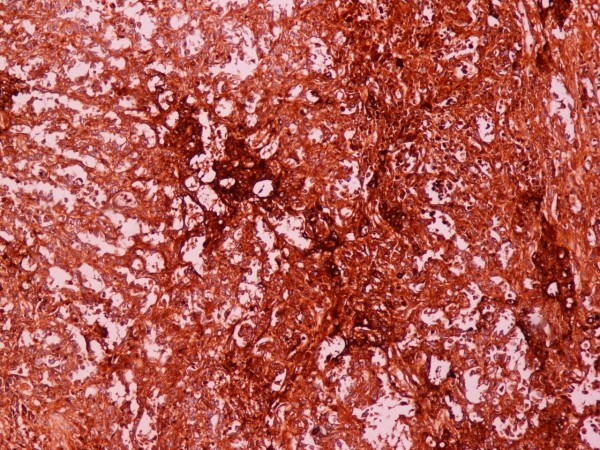
**Yolk-Sac tumor**. AFP ×100.

**Figure 4:**
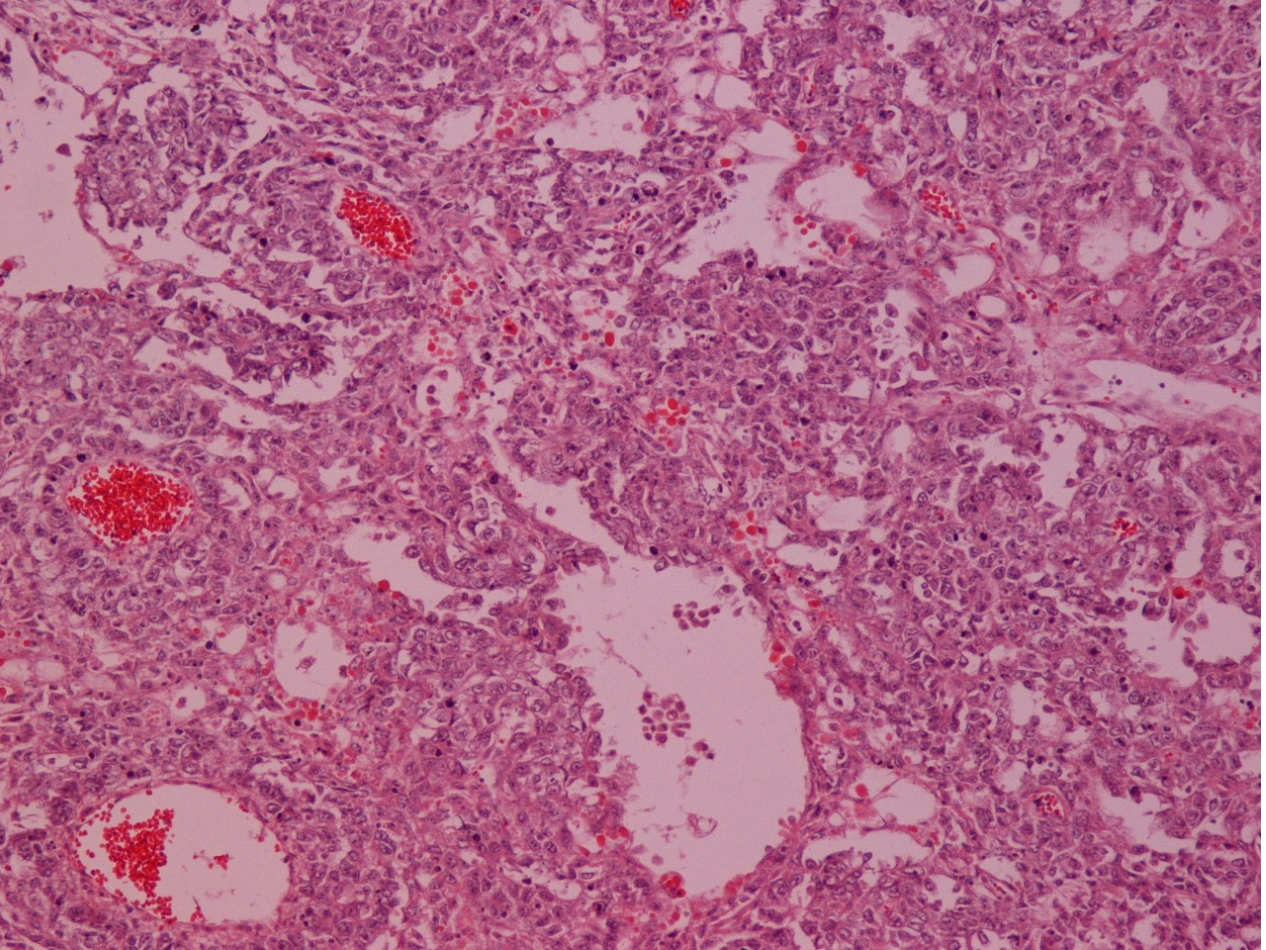
**Seminoma**. H-E ×100.

**Figure 5:**
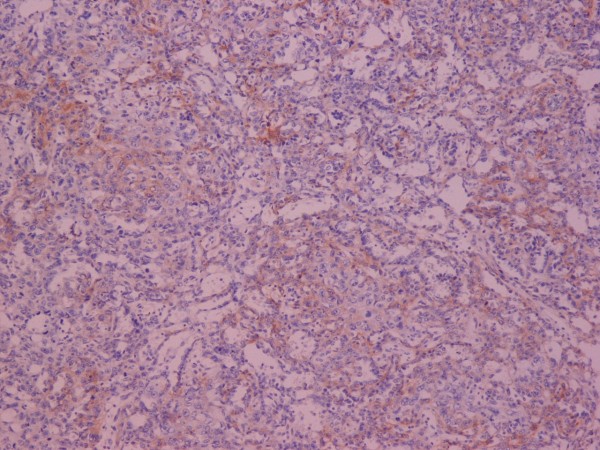
**Seminoma**. c-Kit (CD117) ×100.

**Figure 6:**
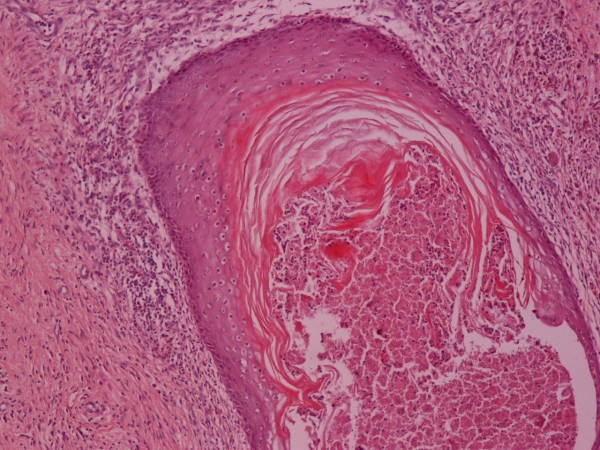
**Keratinizing Squamous Epithelium**. H-E ×100.

**Figure 7:**
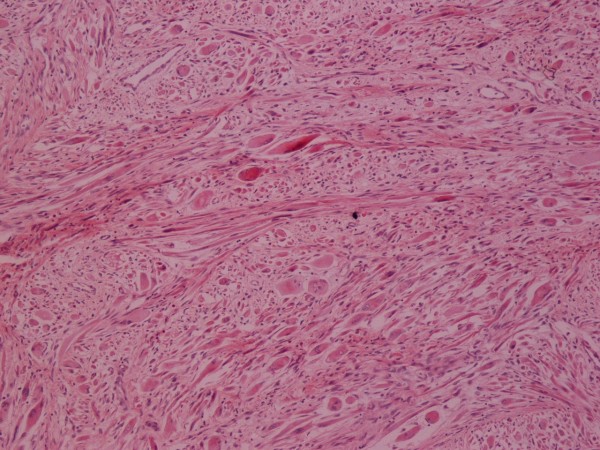
**Rhabdomyosarcoma**. H-E ×100.

**Figure 8:**
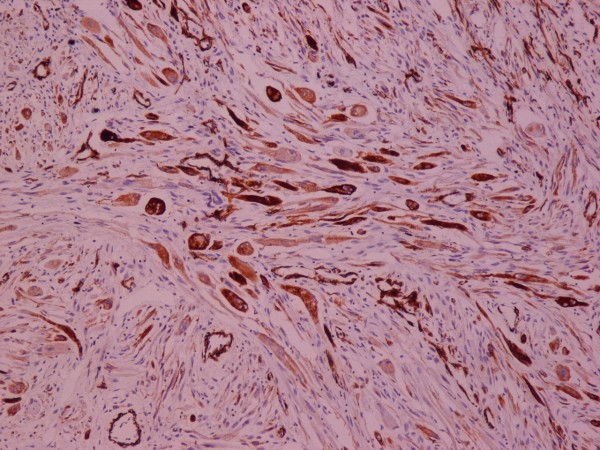
**Rhabdomyosarcoma**. SMA ×100.

On gross pathological examination, there was a, solitary, non encapsulated tumor measuring 4 × 3 × 26 cm located mainly to the originating area of the spermatic cord. The tumor was hard in palpation. On cut section, it had a grating feel and hoary colour. There were areas of hemorrhage and necrosis with yellowish discoloration. Histopathology showed a mixed germ-cell tumor with predominant yolk sac tumor (45%) containing areas of necrosis (figure 3). The tumor it also contained a typical seminoma component (25%) (figures 4 and 5) and immature teratomatous element (30%). The last was consisted of mature keratinizated columned squamous epithelium (figure 6), respiratory epithelium, cartilage and immature rhabdomyoblastic element that was finally diagnosed as rhabdomyosarcoma (figures 7 and 8).

The tumor infiltrated the tunica albuginea. In close proximity to the tunica albuginea a small number of vascular spaces containing neoplasmatic embolus. Epididimis was free of malignant component while diffuse hyperemic spaces were observed along the spermatic cord.

The patient was given chemotherapy of the BEP regimen, i.e. Bleomycin, Etoposide, and Cisplatin. He showed good response after the 1^st ^cycle. At the time of starting the second cycle, AFP had decreased to 98 IU/ml and was accompanied by a moderate reduction of the dimensions of the retroperitoneal mass. However, after the 2^nd ^cycle the patient discontinued the chemotherapy due to personal family problems. When he came for follow-up after a gap of 3 months, he presented with complications of breathlessness, loose motions and loss of weight. Despite the normalisation in tumor markers values, the retroperitoneal mass was relapsed. CT scan of the chest showed multiple lung metastases. After oncology evaluation, the patient was found unfit for further chemotherapy. The poor prognosis was explained to him and to his relatives. He was treated symptomatically. However, finally he succumbed to death after two months due to complications.

## Discussion and conclusion

About 99% of neoplasms of the testis are malignant and they are one of the commonest forms of cancers in young adult males. Two decades ago testis tumors were the most common solid tumor cause of death in young males. Advances in clinical pathology, progress of radiologic and diagnostic testing and the development of novel chemotherapies, allowed for a more accurate staging and treating of testis cancer.

Today, chemotherapy is generally highly effective and achieves some of the highest successes in the field of oncology.

Despite the progress in the diagnosis and treatment, testis cancer continues to present a formidable challenge. In fact, ten percent of patients present with symptoms of metastasis at diagnosis and up to thirty-five percent of patients have evidence of metastatic disease when first seen. It is noteworthy how such an easily accessible and superficially palpable tumor escapes detection till a very late stage of its presentation. The most probable explanation for the delay in diagnosis from the time of initial recognition to the time of treatment is the insidious presentation and lack of overt signs. This delay not uncommonly exceeds six months. It should be noticed however that some testis cancers progress rapidly either because of their rapid growing rate or because of their ability to metastasize. With the exception of spermatocytic seminoma, germ-cell tumor types usually develop retroperitoneal lymph-node metastases -especially NSGCs [4].

A review of the literature revealed a few case reports of mixed germ cell tumor of the testicle with sarcomatous component [5-8]. Theories about its pathogenesis include derivation of the tumor cells from pluripotential germ cells and malignant transformation from teratomatous elements [9]. The last is somehow controversial since testicular teratomas themselves represent the end stage of a differentiation process from other types of malignant germ cell tumor [10]. This concept is being supported by the following observations: Firstly, prepubertal testicular teratomas are benign, yet their morphologically similar counterparts in the postpubertal testis are malignant, whether mature or immature. In fact, postpubertal testicular teratomas have a more disordered arrangement, frequently show significant cytological atypia and may have widespread mitotic activity [10]. Secondly, genetic analysis has shown that the teratomatous elements in postpubertal mixed germ cell tumors of the testis have strikingly parallel allelic losses compared to the nonteratomatous components of the same tumor [10].

Notably, it is very unusual for an embryonal rhabdomyosarcoma to develop on purely teratoma. In addition the sarcomatous element can be present in the primary excision or it can appear after chemotherapy in the metastases [11]. Both facts are indicating that factors other than malignant transformation from teratomatous elements contribute to the development of sarcoma.

Approximately 60% of the patients with NSGC tumors present with advanced clinical disease, i.e. metastasis through the lymphatic as well as hematogenous routes. The most important predictor of metastases is the presence of vascular/lymphatic invasion in the primary tumor and the amount and degree of aggressiveness of a distinct component as well [12]. In fact, about 60% of NSGC tumors are composed of more than one of the pure patterns. The metastasis usually reflects the histology of the basic primary tumor. However, different histologic cell types are found more often in metastases than in primary tumors. This may be due to maturation of one germ-cell type into another cell type. This might not be the case in our patient because of the normalisation in tumor markers values after chemotherapy. To our knowledge, elevated levels of AFP are seen almost exclusively in tumors containing yolk sac elements. AFP is demonstrable in approximately 92% of both primary and metastatic yolk sac tumors. In rare cases, AFP may not be demonstrable in a metastatic viable yolk sac tumor even though the primary tumor is positive for AFP.

In general, 90 to 100% of patients with localized tumors can be cured and up to 70% with metastatic disease can be cured. The therapy and prognosis of testicular tumors can depend largely on clinical stage and on histological type. According to the above criteria our patient would be classified to the intermediate risk group (NSGC tumor, primary location on testis-retroperitoneal space, AFP 1000-10.000, HCG 5000-50.000, LDH > 500, no brain, liver and bone metastases) having an 80% possibility of complete cure. However, the histologic subtype appears to critically influence the prognosis: It is the choriocarcinoma component which is the most aggressive as it spreads rapidly through the hematogenous route, thus leading to a poorer prognosis of patients carrying mixed NSGCs as compared to seminomas [13]. The presence of embryonal carcinoma comprising over 40% of the primary tumor has been also associated with a tendency to metastasize [12]. It is also well documented that the presence of yolk sac elements is highly associated with precocious metastases [14], while accumulative evidence suggests that teratomas with somatic-type malignancies are associated with a poor prognosis [4]. Similarly, the occurrence of sarcomatous elements in germ cell tumors is considering a poor prognostic sign [15]. Actually the development of sarcomas in patients with germ cell tumors is rare and for this reason, in patients with mixed NSGC tumors, metastases composed purely of sarcoma are rare also [5], however, prognosis is usually dependent on the degree of aggressiveness of the sarcomatous component.

In our case, this unusual combination not commonly reported in the literature (yolk sac tumor and immature teratomatous element consisted of rhabdomyosarcoma), appears to have influenced the prognosis.

Another important factor significantly contributing to the definite cure is completion of treatment and regular follow-up of the patient. As demonstrated in our case, any break in the treatment can and will lead to flaring up of the complications and carries very poor prognosis.

## Conclusion

More than 50% of germ-cell tumors include more than 2 basic germ-cell tumor types, with the exception of spermatocytic seminoma. About 90% of the patients with nonseminomatous tumors can achieve complete cure with aggressive chemotherapy and most of them can be cured. Although prognosis of testicular tumors depends largely on clinical stage, histological type and adhesion to the treatment influence the prognosis as well.

## Consent

The authors would like to thank the patient's relatives for providing informed consent for the publication of this case report.

## Competing interests

The authors declare that they have no competing interests.

## Authors' contributions

SK, PP and GP were involved in the case directly. SK drafted the manuscript. NG and MO took part in the care of the patient PP contributed in the preparation of the manuscript. TV contributed in carrying out the medical literature search. SK was involved in conception of the article and revising it critically for important intellectual data before final approval. All authors reviewed the final drafting of this manuscript.
